# Geological Hazard Susceptibility Analysis and Developmental Characteristics Based on Slope Unit, Using the Xinxian County, Henan Province as an Example

**DOI:** 10.3390/s24082457

**Published:** 2024-04-11

**Authors:** Wentao Yang, Ruiqing Niu, Rongjun Si, Jun Li

**Affiliations:** 1 College of Resources and Environment, Henan Polytechnic University, Jiaozuo 454003, China; 212103010028@home.hpu.edu.cn; 2 School of Geophysics and Geomatics, China University of Geosciences, Wuhan 430074, China; rqniu@163.com (R.N.); junl@cug.edu.cn (J.L.)

**Keywords:** susceptibility of geological hazard, geological hazard, slope unit, random forest, analytic hierarchy process, information method

## Abstract

Geological hazards in Xinxian County, Xinyang City, Henan Province, are characterized by their small scale, wide distribution, and significant influence from regional tectonics. This study focuses on collapses and landslide hazards within the area, selecting twelve evaluation factors: aspect, slope shape, normalized difference vegetation index (NDVI), topographic relief, distance from geological structure, slope, distance from roads, land use cover type, area of land change (2012–2022), average annual rainfall (2012–2022), and river network density. Utilizing data from historical disaster sites across the region, the information quantity method and hierarchical analysis method are employed to ascertain the information quantity and weight of each factor. Subsequently, a random forest model is applied to perform susceptibility zoning of geological hazards in Xinxian County and to examine the characteristics of these geological disasters. The results show that in the study area, the primary factors influencing the development of geohazards are the distance from roads, rock groups, and distance from geological structure areas. A comparison of the susceptibility results obtained through two methods, the analytic hierarchy process information quantity method and the random forests model, reveals that the former exhibits a higher accuracy. This model categorizes the geohazard susceptibility in the study area into four levels: low, medium, high, and very high. Notably, the areas of very high and high susceptibility together cover 559.17 km^2^, constituting 35.99% of the study area’s total area, and encompass 57 disaster sites, which represent 72.15% of all disaster sites. Geological hazards in Xinxian County frequently manifest on steep canyon inclines, along the curved and concave banks of mountain rivers, within watershed regions, on gully inclines, atop steep cliffs, and on artificially created slopes, among other sites. Areas with very high and high vulnerability to these hazards are mainly concentrated near the county’s geological formations. The gneiss formations are widely exposed in Xinxian County, and the gneisses’ strength is significantly changed under weathering, which makes the properties of the different degrees of weathering of the rock and soil bodies play a decisive role in the stability of the slopes. This paper provides a basis for evaluating and preventing geologic hazards in the Dabie mountainous area of the South Henan Province, and the spatial planning of the national territory.

## 1. Introduction

In China, geologic disasters are among the most severe worldwide, leading to significant economic losses, casualties, and hindrances to local socio-economic development [1,2]. The country’s intricate regional geological conditions, coupled with recent extreme weather events, have escalated the frequency of various geologic hazards [3,4]. A planned and systematic approach is essential for the rational development and utilization of land resources, focusing on the prevention and control of geological hazards. Methodologies such as investigating molecules, assessing and forecasting geological hazards in specific areas, and creating geological hazard susceptibility zoning maps serve as critical references for urban infrastructure planning. These strategies are vital for mitigating the impacts of geologic hazards before their occurrence [5].

Currently, widely employed methods for assessing susceptibility to geological hazards encompass statistical approaches, such as the deterministic coefficient, weight-of-evidence, informativeness, support vector machine, fuzzy judgment, and linear regression analysis methods, and empirical models, such as the expert scoring and hierarchical analysis methods. Machine learning techniques, such as random forest and artificial neural networks, are also employed [6,7,8,9]. Empirical models, which rely on subjective judgment, tend to produce results overly dependent on expert knowledge, thus lacking objectivity. Conversely, statistical models often overlook the intricate relationships among factors and are best suited for smaller areas. Integrating empirical models with statistical approaches can yield more scientifically robust and accurate evaluations of geological hazard susceptibility [10,11,12,13,14,15].

Among the prevalent machine learning models, the support vector machine (SVM) often overfits when feature count substantially exceeds the sample size, and it also lacks direct weight provision and faces challenges in visualizing data in high dimensions [16,17,18]. In contrast, the logistic regression model typically underperforms, with generally low accuracy [19,20,21], while the random forest algorithm, benefiting from low data requirements and gradient boosting, mitigates overfitting to a degree [22,23,24,25]. The application of random forest (RF), SVM, and naive bayes (NB) methods in constructing a geohazard susceptibility assessment model for the Puge section of the Zemu River Valley in the Liangshan Yi autonomous prefecture has demonstrated superior RF model accuracy [26]. The random forest model was leveraged to derive objective weights and to integrate information, which significantly enhanced the accuracy and the reliability of geohazard susceptibility mapping over traditional methods in Kang County, Gansu Province, [27]. The ratio of positive to negative geohazard samples for assessing susceptibility in Liulin County with the RF model revealed optimal performance at a 1:5 sample ratio [28]. Model prediction accuracy escalated with sample size up to a critical threshold, beyond which it diminished. The logistic regression, random forest, boosted regression tree (BRT), and combined BRT-LR and BRT-RF models for susceptibility analysis and validation showcase the BRT-RF model’s efficacy in enhancing the accuracy of landslide susceptibility (LS) prediction [29,30].

Xinxian County, situated in the heart of the Dabie Mountains in southern Henan’s low mountain and hilly region, is prone to landslides, avalanches, and other geological disasters, with an exceptionally high disaster development density. In this county, geological disasters are primarily associated with human engineering activities. The rapid urbanization in Xinxian County’s urban areas, combined with unique local geographic conditions, has lead to the creation of numerous unstable artificial slopes. These are the results of mountain excavation and ditch filling to expand residential spaces. Moreover, the complex geological phenomena in Xinxian County, driven by significant tectonic activity, have altered the rock structure, with joints and fissures in rock layers being well-developed, thereby facilitating geological disasters. Notably, gneiss, a type of metamorphic rock highly susceptible to geological disasters, predominates in the area. Its exposure on the surface results in the development of joints and fissures due to tectonic forces, and loosening structure after weathering. When these factors combine with human activities and rainfall, the risk of geological disasters is significantly increased. The use of the information quantity model and hierarchical analysis method in evaluating geological hazard susceptibility suffers from the influence of subjective factors, leading to less accurate and less objective evaluation results.

This study designated Xinxian County as the research area, drawing on field survey data and previous research to analyze the geological environment and disaster characteristics therein. It identified critical geological disaster evaluation factors, incorporating the land change area to more precisely assess the impact of human engineering activities on local geological disaster development. This study assessed and classified geological hazard susceptibility using the analytic hierarchy process (AHP), the information quantity method, and random forest model. It further investigated the development patterns of local geological disasters and provided an in-depth analysis of disaster characteristics within the principal rock exposure zones of Xinxian County.

## 2. Overview of the Study Area

### 2.1. Geological Overview of the Study Area

**Figure 1:**
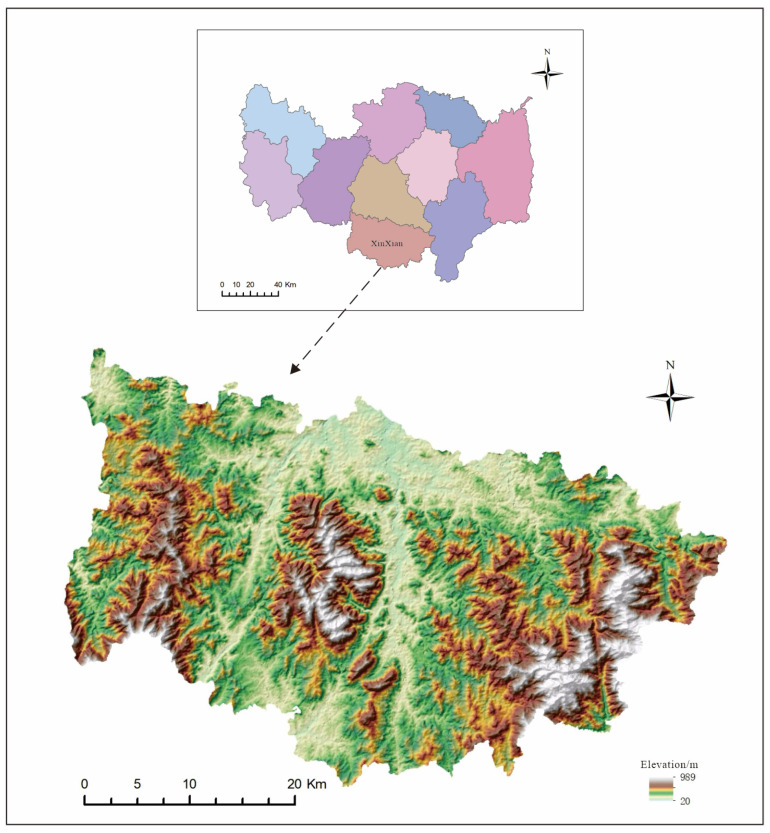
Schematic of the location of the study area.

Xinxian County, a part of Xinyang City in the Henan Province, is situated in the heart of the Dabie Mountains in southern Henan. It serves as a junction among six counties across the Hubei, Henan, and Anhui Provinces. The county spans 61.6 km east to west and 40.7 km north to south, covering a working area of 1556 square kilometers (Figure 1).

The geographic coordinates of Xinxian County are between 114°32′ to 115°13′ east longitude and 31°27′ to 31°50′ north latitude. The county seat is situated 150 km from Xinyang City, benefiting from efficient transportation networks, including the Daguang Expressway and the 106 National Highway. These main arteries interlink six major roads, forming a comprehensive network that connects all townships and administrative villages within the county by car. Geographically, the county straddles two significant watersheds: the Yangtze River Basin and the Huaihe River Basin. Of its six major water systems, the Dui, Bailu, Zhugang, and Zhai Rivers are parts of the Huaihe River Basin, while the Panshui and Lushui Rivers feed into the Yangtze River Basin.

Xinxian County’s geological formations encompass the Paleoproterozoic Dabie rock group, the middle Neoproterozoic Huwan and Dingyuan rock formations, the Paleozoic Nanwan formation, and Neoproterozoic to Holocene strata of the quaternary period. The predominant rock and soil types are hard massive and intrusive rock formations, harder massive gneiss formations, thinly laminated quartz schist formations, and river valley stacked loose rock types. These formations exhibit a block structure and are dense and complex, with high compressive strength ranging from 31 to 52.0 MPa. The weathering zone typically spans 2–5 m in thickness, extending locally to 6–10 m. Most gneiss exposed on the surface within the surveyed area show a high degree of weathering, with the development of joints and fissures, a loose structure, and the tendency to crumble easily when handled.

### 2.2. Data Sources

The information on geo-hazard sites was obtained from the data of the Xinxian 1:50,000 Geological Hazard Detailed Survey Project. Geography-related data were obtained from Xinxian 1:50,000 mapping data, geological structure-related data were obtained from Xinxian 1:50,000 geological map, and the 12.5 m Digital Elevation Model (DEM) was obtained using the Advanced Land Observing Satellite-2 (ALOS-2) data available from the National Aeronautics and Space Administration (NASA) (https://search.asf.alaska.edu/ (accessed on 9 March 2024)). Remote sensing image data from Land-sat 8 Satellite Digital Product were obtained from Geospatial Data Cloud (https://www.gscloud.cn/ (accessed on 10 March 2024)). Vegetation cover was calculated by Landsat 8-Oli data, downloaded from Geospatial Data Cloud. Land use data were obtained from the Resource and Environment Science and Data Center of the Chinese Academy of Sciences (https://www.resdc.cn/ (accessed on 9 March 2024)). Average annual rainfall and river network density data were obtained from the National Earth System Science Data Center (http://www.geodata.cn (accessed on 9 March 2024)), and road data were obtained from Sky Map (https://www.tianditu.gov.cn/ (accessed on 9 March 2024)). For data processing, advanced software packages, including ENVI 5.6 and ArcGIS, were utilized. Factors possessing varying spatial resolutions were resampled to acquire the necessary evaluation factors for assessing geological hazard susceptibility in Xinxian County (Table 1).

## 3. Methods

### 3.1. Slope Unit Classification

**Figure 2:**
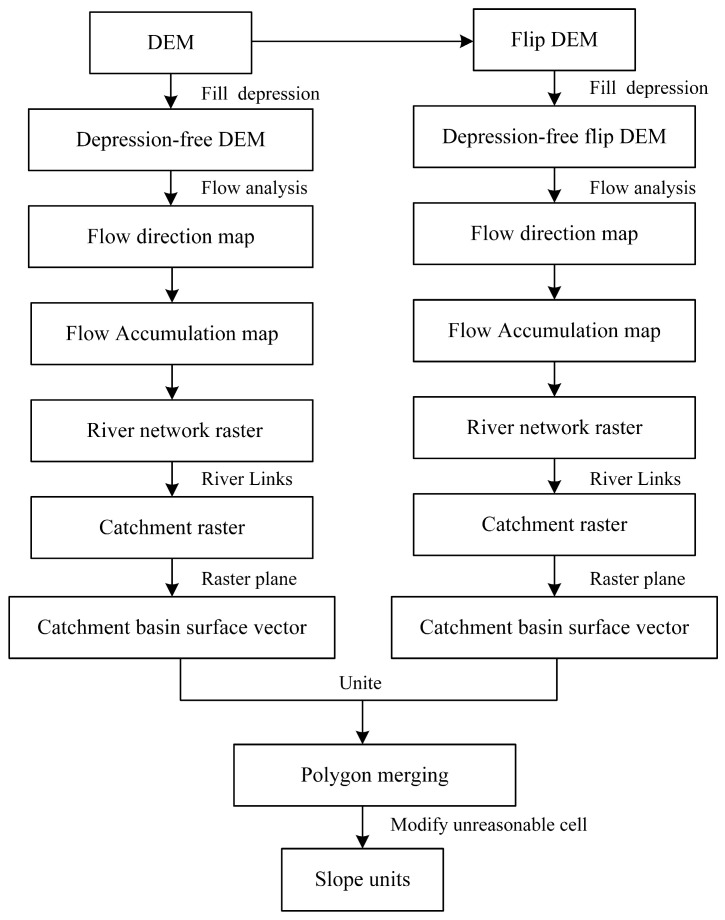
Flowchart of slope unit extraction by hydrologic analysis method.

The slope unit is identified as the fundamental topographical entity for landslide analysis. Utilizing slope units as evaluative entities mitigates the shortcomings inherent to traditional raster units, which often compromise the delineation of slopes, thus enhancing the representation of the topographic and geomorphic characteristics of the study area. Among numerous other factors, the developmental stage of river valleys profoundly influences the frequency of landslides and avalanches. By classifying valleys as young, slope units can be effectively aligned with the geo-environmental conditions of the area. Considering various influencing factors ensures that evaluation outcomes more closely mirror actual conditions. This study employed the hydrological analysis method, specifically utilizing the ArcGIS hydrological analysis tool to manipulate the Digital Elevation Model (DEM). This process involved filling in the depressions to delineate ridgelines and valley lines, merging the resultant catchment areas with their inverses, and subsequently refining the slope units through manual adjustments [31] (Figure 2).

### 3.2. Information Quantity Method

Historically, the information quantity method is predominantly utilized in geological prospecting and is adapted to assess geohazards. This approach involves converting measured values that indicate various factors affecting regional stability into informativeness values. These values are designed to reflect regional stability by incorporating both actual occurrences and data from past geohazards [32,33]. The evaluation process calculates the informational content related to the factors influencing the study subject. Specifically, it quantifies the impact of these factors on geological hazard susceptibility by determining the information quantity they represent [34].

In the case of a single evaluation factor, the method of evaluating the information quantity of the unit is:

(1) $$I_{x_{i}}=Ln\frac{N_{i}/N}{S_{i}/S}$$

where $\;I_{x_{i}}\;$ is the evaluation factor, $x_{i}$ is the amount of information provided by the evaluation unit, $N_{i}$ is the total number of geohazards distributed within a specific level within the evaluation factor $x_{i}$, $N_{i}$ is the number of evaluation units containing the evaluation factor *x_i_*, *N* is the total number of geohazards, and *S* is the total number of the evaluation unit [35].

### 3.3. Analytic Hierarchy Process

The Analytic Hierarchy Process (AHP), a decision-making method introduced by American operations researcher T.L. Saaty in the 1970s, is widely used for evaluating geohazard susceptibility. This method adeptly illustrates the degree of interconnection among various factors. The process involves four main steps: establishing a hierarchy, constructing a judgment matrix, determining the weights of indicators, and testing the judgment matrix’s consistency [36,37,38].

In this study, geohazard susceptibility serves as the target layer, which is further decomposed into the guideline layer and the factor layer. Hazard-causing factors within the study area are categorized into regional geologic factors and ecological and anthropogenic factors. Weights for these factors are determined using a formula derived from the hierarchical analysis model established for this purpose.

(2) $$w_{i}=\frac{1}{n}\sum_{j=1}^{n}\frac{a_{ij}}{\sum_{k=1}^{n}a_{kj}}\left(i=1,2,3,\ldots ,n\right)$$

(3) $$\lambda_{max}=\sum_{i=1}^{n}\frac{({Aw)}_{i}}{nw_{i}}$$

where w is the eigenvector (weight), n is the order of the judgment matrix, $a_{ij\;}$ is the result of the comparison of the importance of the influence factor i and the influence factor j, $a_{ij\;}$ constitutes the judgment matrix *A*, $\sum_{k=1}^{n}a_{kj}$ is the step of the judgment matrix *A* summing by columns, which is an essential step in the method of normalization by columns used in the search for the eigenvector w, and *λ_max_* is the maximum eigenvalue.

The consistency test formula for judgment matrices is as follows:

(4) $$CI=\frac{\lambda_{max}-n}{n-1}$$

(5) $$CR=\frac{CI}{RI}$$

where CI is the consistency index, CR is the ratio of one-coherence, and RI is the average randomness index. Usually, whether CR is less than 0.1 is used as a criterion for the satisfaction of the judgment matrix; if CR < 0.1, then it indicates that the judgment matrix consistency is better [39].

### 3.4. Random Forest

The random forest (RF) model, a machine learning algorithm, incorporates multiple decision trees and falls under the bagging category. It achieves highly accurate and generalized classification results by aggregating multiple weak classifiers through voting or averaging. The RF model boasts rapid training speed, exceptional accuracy, the capacity to process high-dimensional data, and robust adaptability to the training set [40,41,42].

When constructing a decision tree as part of a random forest model, each tree is associated with its own training set. To construct K decision trees, K distinct training sets are required. During training set creation, samples are randomly selected from the complete dataset using a method known as “random and put-back” sampling, resulting in a total of S training subsets. The ultimate model output is determined by a voting mechanism, where the classification outcome receiving the majority of votes becomes the algorithm’s final output [43].

## 4. Results

### 4.1. Slope Unitization

**Figure 3:**
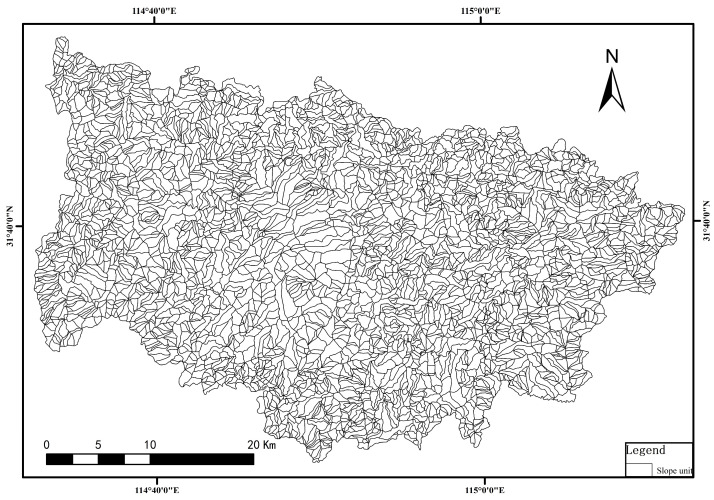
Results of slope unit division in the study area.

By employing the hydrological analysis method within ArcGIS to analyze the Digital Elevation Model (DEM) of Xinxian County, slope units were delineated based on four different threshold values: 500, 1000, 2000, and 3000. Upon comparison, the slope units defined at the threshold of 500 exhibited the highest consistency with on-field observations. Subsequent manual adjustments were made to refine these delineations, resulting in a total of 4036 slope units (Figure 3).

### 4.2. Evaluation Indicator Factor Selection

**Figure 4:**
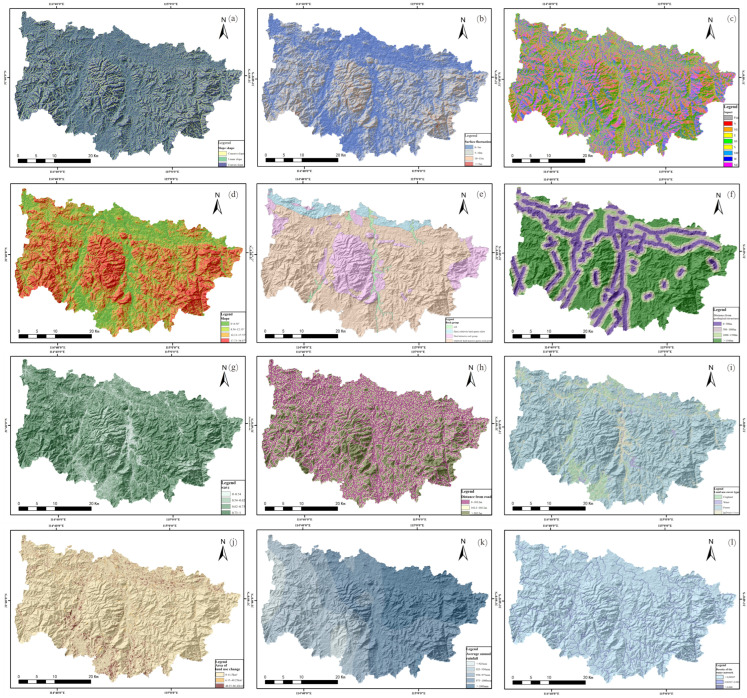
Distribution of geohazard susceptibility evaluation factors ((**a**) Slope shape; (**b**) Topographic relief; (**c**) Aspect (**d**) Slope; (**e**) Rock group; (**f**) Distance from geological structure; (**g**) NDVI; (**h**) Distance from road; (**i**) Land use cover type (**j**) Area of land use change; (**k**) Average annual rainfall; (**l**) River network density).

This study undertook a detailed analysis of disaster breeding conditions, the geological environment, and the distribution patterns of geological disasters in Xinxian County. Through a review and comparison with factors selected in previous research, twelve factors were chosen for evaluation: slope shape (the curvature of a slope), NDVI, topographic relief (the vertical and horizontal dimensions of land surface), distance from geological structure, slope, distance from the road, aspect, land use cover type, area of land change (2012–2022), average annual rainfall (2012–2022), and river network density. Each evaluation index underwent quantitative classification. The resolution of the evaluation factors’ raster images was set at 12.5 m × 12.5 m, dividing the study area into a total of 9,948,409 raster units. The information quantity model calculated the information value for each evaluation factor, assessing their impact on the occurrence of geohazards within the study area (Figure 4).

### 4.3. Analytic Hierarchy Process to Calculate Factor Weights

#### 4.3.1. Analytic Hierarchy Process Method Structural Model

**Figure 5:**
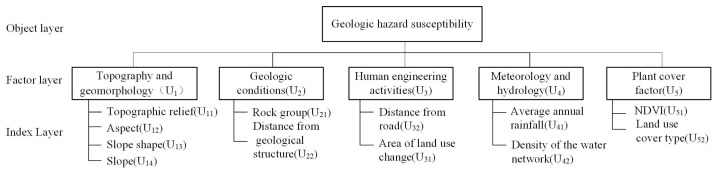
Analytic hierarchy process method structural model.

The assessment framework was organized into three tiers: the object layer, the index layer, and the factor layer. At the core, the object layer focused on geohazard susceptibility (U), branching into five index layers: topography and geomorphology (U_1_), geological conditions (U_2_), human engineering activities (U_3_), meteorology and hydrology (U_4_), and plant cover (U_5_). Each index layer was further dissected into factor layers as follows: topography and geomorphology encompasses topographic relief (U_11_), aspect (U_12_), slope shape (U_13_), and slope (U_14_); geological conditions were detailed by rock group (U_21_) and distance from geological structure (U_22_); human engineering activities included distance from road (U_31_) and area of land use change (U_32_); meteorological and hydrological factors consisted of average annual rainfall (U_41_) and river network density (U_42_); and the plant cover factor comprised the normalized vegetation index (NDVI) (U_51_) and land use cover type (U_52_) (Figure 5).

#### 4.3.2. Analytic Hierarchy Process Method Weight Calculation

The judgment matrices for each criterion level evaluation factor were:

U−U_i_ standardized layer: $\left(\begin{array}{cc} \begin{array}{ccc} 1 & 1/5 & 1/4\\ 5 & 1 & 2 \end{array} & \begin{array}{cc} 1/3 & 1/2\\ 2 & 3 \end{array}\\ \begin{array}{ccc} \begin{array}{c} 4\\ 3 \end{array} & \begin{array}{c} 1/2\\ 1/2 \end{array} & \begin{array}{c} 1\\ 1/2 \end{array}\\ 2 & 1/3 & 1 \end{array} & \begin{array}{cc} 2 & 1\\ \begin{array}{c} 1\\ 1/2 \end{array} & \begin{array}{c} 2\\ 1 \end{array} \end{array} \end{array}\right)$;

U_1_–U_1j_ standardized layer: $\left(\begin{array}{cc} \begin{array}{cc} 1 & 1/2\\ 2 & 1 \end{array} & \begin{array}{cc} 1/2 & 1/2\\ 1 & 1 \end{array}\\ \begin{array}{cc} 2 & 1\\ 2 & 1 \end{array} & \begin{array}{cc} 1 & 1/2\\ 2 & 1 \end{array} \end{array}\right)$;

U_2_–U_2j_ standardized layer: $\left(\begin{array}{cc} 1 & 3\\ 1/3 & 1 \end{array}\right)$;

U_3_–U_3j_ standardized layer: $\left(\begin{array}{cc} 1 & 1/3\\ 3 & 1 \end{array}\right)$;

U_4_–U_4j_ standardized layer: $\left(\begin{array}{cc} 1 & 1/2\\ 2 & 1 \end{array}\right)$;

U_5_–U_5j_ standardized layer: $\left(\begin{array}{cc} 1 & 1/2\\ 2 & 1 \end{array}\right)$.

After calculation, λ_max_ = 5.15 and consistency index CR = 0.0337 for the object layer (U) and factor layer (U_i_); λ_max−1_ = 4.06 and CR = 0.0227 for the factor layer (U_i_) and index layer (U_ij_); λ_max−2_ = 2 and CR = 0; λ_max−3_ = 2 and CR = 0; λ_max−4_ = 2 and CR = 0; and λ_max−5_ = 2, CR = 0. Upon comparison, it was known that the consistency index CR of the judgment matrix was <0.1, and normalization was performed to obtain the weight value of each evaluation factor (Table 2).

### 4.4. Evaluation of the Informativeness Model

Various evaluation factors were derived through the utilization of ArcGIS for regional analysis. The informational value of these factors across different levels was determined by overlaying them with 79 geohazard points, followed by a comprehensive generalization and analysis of each factor. A higher information quantity value indicated an increased likelihood of geohazard occurrences. Consequently, the evaluation factors were categorized based on intervals that signified a favorable probability for geohazard events.

#### 4.4.1. Topography and Geomorphology

Topographic and geomorphological factors served as the primary determinants for the distribution of geohazards, which were predominantly located on convex slopes. In contrast, concave slopes exhibited a reduced susceptibility to such hazards. Geohazards demonstrated a notably stabilized presence within areas experiencing topographic relief between 5 and 10 m, and slopes ranging from 8.54 to 12.13 degrees. Furthermore, the distribution of slope aspects was varied, with a heightened probability of geohazard occurrence on slopes facing southwest and south.

#### 4.4.2. Geologic Conditions

Hard and relatively hard quartz schist, including dolomitic diorite and hornblende gneiss, demonstrate lower compressive strength and undergo a higher degree of weathering, consequently increasing the likelihood of geological hazard occurrences. Such hazards were most commonly initiated on weak structural surfaces proximate to fracture zones, which significantly influenced the susceptibility to geological hazards. Furthermore, the proximity of geological structures was directly associated with a heightened frequency of collapses and landslides, as evidenced in Table 3.

#### 4.4.3. Human Engineering Activities

The study area was predominantly characterized by mountainous and hilly terrain. Human engineering activities, including road construction and house building, had altered the internal stress state of slope bodies, thereby increasing the propensity for rock body collapses and the induction of geological disasters. The extent of land use change served as an indicator of the intensity of human engineering activities. Proximity to highways was directly correlated with an increased likelihood of geohazards. Furthermore, medium-sized changes in land use significantly affected the occurrence of geohazards.

#### 4.4.4. Meteorology and Hydrology

The groundwater within the study area primarily consisted of bedrock fissure water, supplemented by a minor proportion of terrace valley loose rock fissure water. This bedrock fissure water was profoundly influenced by geological structures, resulting in limited connectivity and anisotropic hydraulic interactions. Typically, extended periods of rainfall and heavy downpours significantly increased the likelihood of triggering landslides and other geological hazards. Areas characterized by a river network density ranging from 0.00267 to 0.080, receiving an average annual rainfall between 925 and 950 mm, were particularly susceptible to geological hazards.

#### 4.4.5. Plant Cover Factor

Vegetation cover significantly influenced slope stability; increased vegetation coverage reduced the impact of precipitation infiltration on slopes. In the study area, geological disasters within regions exhibiting an NDVI (Normalized Difference Vegetation Index) between 0 and 0.73 constituted 88.6% of all geological disasters. This revealed a negative correlation between dense vegetation and the likelihood of geological disasters, indicating that areas with denser vegetation were less prone to such disasters. Moreover, geological disasters tended to occur more frequently on lands designated for construction.

**Figure 6:**
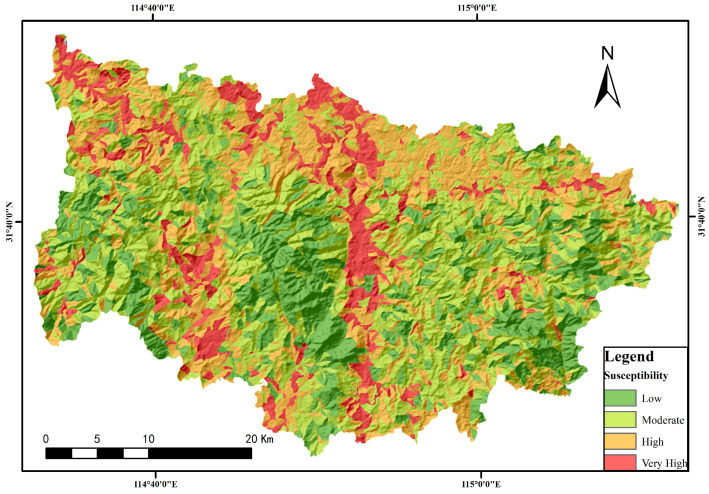
AHP-information quantity model of Xinxian County susceptibility distribution based on slope unit.

Based on the chosen evaluation factors and their respective weights, as determined through the hierarchical analysis method, and taking into account the geologically predisposed conditions and known geohazard locations within the study area, a weighted analysis of each factor was performed to compute the area’s comprehensive weighted information. The natural break-point method facilitated the classification of geohazard susceptibility into four distinct categories: very high, high, medium, and low susceptibility. This classification process yielded a geohazard susceptibility evaluation map, visualized on a raster basis (refer to Figure 6). 

### 4.5. Random Forest Modeling

This study simplified landslide occurrence into a binary classification issue. Locations of geological hazards were identified as positive samples (labeled as “1” in the dataset), signifying areas where such events transpired. Conversely, sites without geohazards or landslides were classified as negative samples (labeled as “0”), denoting regions unaffected by landslides.

**Figure 7:**
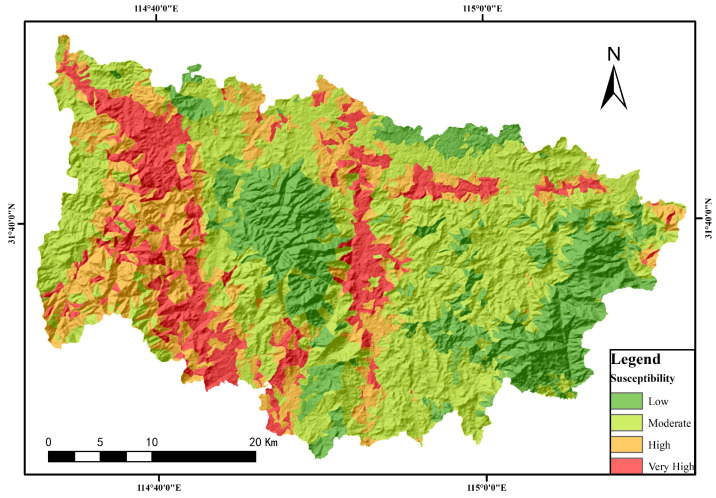
Stochastic model based on slope unit for the distribution of geohazard susceptibility in Xinxian County.

The random forest algorithm’s hyperparameters encompass “n_estimators”, denoting the total count of decision trees within the ensemble. Typically, an increased number of trees enhances the model’s performance by bolstering its robustness and diminishing the likelihood of overfitting. “Max_depth” refers to the decision trees’ maximum allowable depth, where excessively high settings may induce overfitting and overly low values could lead to underfitting. The “criterion” function assesses feature selection quality, with the quantity and depth of decision trees determining their fitting accuracy. Widely utilized metrics for this evaluation include information gain and the Gini coefficient. The selection of optimal parameters was informed by an analysis of pertinent literature and an examination of data specific to the research area, as elaborated in Table 4. The geologic hazard susceptibility map is shown in Figure 7.

## 5. Evaluation Findings and Analyses

### 5.1. Results of the Susceptibility Assessment

#### 5.1.1. AHP-Information Quantity Model Susceptibility Results

The geological disaster susceptibility zoning results for Xinxian County indicated that the extents of the low, medium, high, and very high susceptibility zones were 193.15 km^2^, 366.02 km^2^, 607.32 km^2^, and 386.97 km^2^, respectively. These zones represent 12.43%, 23.56%, 39.09%, and 24.91% of Xinxian County’s total area, respectively. The distribution of disaster points within these zones were 33, 24, 15, and 7, respectively. Notably, the very high susceptibility zones exhibited a moderate placement and a relatively high density of disaster points, affirming the evaluation’s reasonableness and reliability (refer to Table 5).

#### 5.1.2. Random Forest Model Susceptibility Results

The random forest model’s susceptibility analysis revealed that the low, medium, high, and very high susceptibility zones in Xinxian County covered areas of 194.45 km^2^, 336.39 km^2^, 635.34 km^2^, and 387.28 km^2^, respectively. These zones constituted 12.51%, 21.65%, 40.90%, and 24.93% of the county’s total area, respectively, encapsulating 30, 21, 19, and 9 disaster points, respectively (Table 6).

#### 5.1.3. Comparison

**Figure 8:**
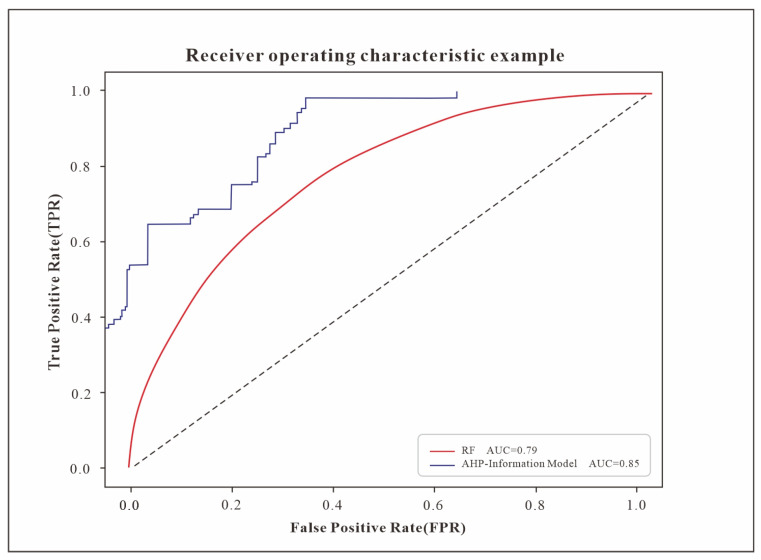
ROC graph.

The receiver operating characteristic curve (ROC curve) [44,45,46] is a crucial metric for evaluating a model’s performance, with the area beneath the curve (AUC value) acting as a direct measure of model accuracy. In the assessment of geological disaster susceptibility in Xinxian County, the AUC values for the random forest and AHP-information quantity models stood at 0.79 and 0.84, respectively. This demonstrated the AHP-information quantity model’s superior precision over the random forest model. Notably, the areas identified as very high risk by the AHP-information Quantity method and the random forest model accounted for 12.43% and 12.52% of the total area, respectively, with disaster point densities of 0.1709 and 0.1543. Despite a similar proportion of areas being classified as very high risk, the AHP-information quantity model reported a higher density of disaster points, indicating a more logically distributed pattern in its susceptibility zoning results (Figure 8).

### 5.2. Characterization of Geohazard Development

#### 5.2.1. Topography and Human Engineering Activities

Steep canyon banks, concave riverbanks in mountainous regions, watershed areas, gully banks, mountainous cliffs, and artificially steepened slopes represented locations with a heightened susceptibility to geological hazards. The vulnerability of these areas has been significantly exacerbated by intensive human engineering activities, particularly in regions constrained by topographical features, and by urban and rural development at the base of slopes. Such activities have compromised slope stability, precipitating the frequent occurrence of geological hazards. In low mountainous terrains, where gullies were prevalent and notable differences in slope and elevation existed, conditions were conducive to the genesis of geological disasters. However, the limited extent of human engineering in these areas resulted in a reduced incidence of such disasters. Conversely, the terrain of river valley terraces, characterized by minimal elevation variation, presented fewer opportunities for the emergence of geological disasters such as avalanches and landslides, resulting in their sparse distribution.

#### 5.2.2. Characteristics of Geological Hazard Development under the Influence of Tectonics

Geological structures, encompassing faults, weak interlayers, zones of tectonic extrusion and compression, misaligned strata, joints, fissures, and disadvantageous combinations of structural surfaces, played a pivotal role in the occurrence of geological hazards. The extent of hazard development was governed by various factors, including the degree of development, scale, interconnectivity, the extent of filling, the composition of fill materials, and the exposure of structural surfaces. In Xinxian County, the majority of geological hazards were located within 500 m of such structures. These hazards predominantly aligned with the nearly east–west and north–south oriented fracture groups, as well as the northwest and northeast orientations. Significant areas of concern included the back slope of Tiantai Mountain, the composite slope of the Baimashan–Xizhuangdian region, and the dome of Baiyun Mountain, all of which are significantly influenced by the region’s geotectonic structure.

#### 5.2.3. Impact of Rainfall on the Development of Geologic Hazards

In the study area, precipitation levels were notably high. This abundance of rainfall led to the rapid formation of slope surface flow, which significantly eroded and undermined loose materials on the slope. Similarly, gully streams, also a result of precipitation, swiftly eroded the base of the slope, creating hollow areas that contributed to rapid slope destabilization and the subsequent onset of avalanches and landslides. Furthermore, the infiltration of atmospheric precipitation could have exacerbated the situation by increasing the weight of the slope’s material without altering the slope gradient. Consequently, an increase in the weight of the loose material on the slope resulted in enhanced downward pressure, further diminishing slope stability.

#### 5.2.4. Development Characteristics of Geologic Hazards in the Gneiss Area of Xinxian County

The gneiss formation encompassed 66.9% of the exposed rock and soil regions within Xinxian County, with its presence spanning all townships and urban areas in the county. The predominant locations for human engineering projects, economic activities, and settlements were situated within the gneiss-dominant areas. This distribution is primarily due to the topographical nature of Xinxian County, which is characterized by hilly and mountainous terrain, limiting the availability of suitable land for construction. As a result, extensive excavation of slopes was necessitated for the development of infrastructure such as roads, residential buildings, and industrial facilities.

**Figure 9:**
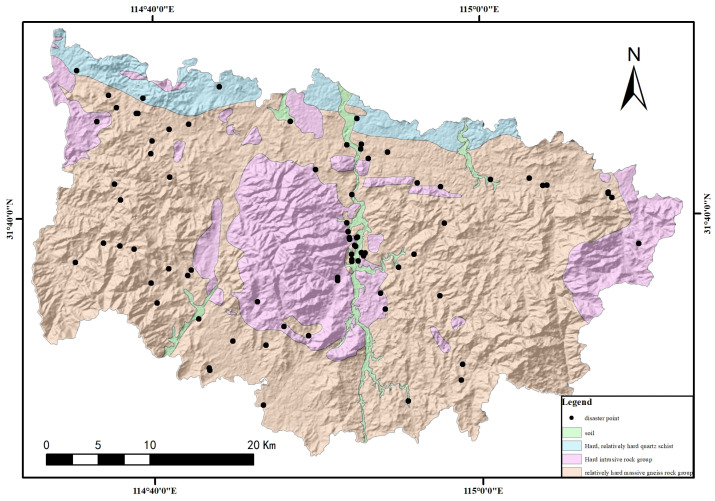
Distribution of geologic hazard sites and rock groups in new county.

The survey area contained 79 significant geological hazards, all categorized as landslides and avalanches, comprising 37 landslides and 42 avalanches. Analysis of the distribution of geotechnical and geological hazards in Xinxian County revealed that these hazards predominantly occur in intrusive rock formations, gneiss rock formations, and areas with loose rock formations. Specifically, gneiss formations were the site for 53 of these hazards, including 21 landslides and 32 avalanches. Geologic hazards and potential risks were presented in gneiss formation areas across all townships and urban areas within Xinxian County. Of the four main types of rock formations identified across the county, hazards located within the gneiss formations represented 67.09% of the total geological hazards and risks identified in the survey, amounting to approximately two-thirds of all cases (Figure 9).

In the newly surveyed area of the county, most of the surface-exposed gneiss exhibited a high degree of weathering. Engineering and construction activities, often influenced by tectonic forces, led to the steep cutting of gneiss slopes, further exacerbated by the development of joints and fissures. This weathering process resulted in loosened structural integrity. When combined with specific material conditions, such as a substantial thickness of completely weathering residual slope layers, or the presence of structurally weak rock and soil surfaces, these areas became highly prone to geological hazards. This susceptibility was particularly heightened by prolonged or intense rainfall, human activities, and other external factors, leading to the formation of shallow surface geological hazards, as detailed in Table 7.

## 6. Discussion

Assessing geological disaster susceptibility through qualitative, quantitative, or a blend of methods traditionally relies on prior experience, potentially introducing inaccuracies in outcomes [14,16]. Machine learning models notably diminish subjective bias in such evaluations [17]. This study enhanced factor weight accuracy by thoroughly assessing geological environmental conditions alongside human impact within the study area. When juxtaposed with the random forest model’s outcomes, both models exhibited similar disaster grade distributions, with extremely high-risk zones constituting 12.43% and 12.52% of the area, and the models returning hazard densities of 0.1709 and 0.1543, respectively. Notably, the AHP-information model revealed a comparable extent of extremely high-risk zones, but a greater density of disaster points, suggesting superior precision in its evaluation results. Hence, the factor weights derived from this study offer valuable insights for assessing geological disasters locally.

The advancement of economic and social development has significantly escalated human engineering activities, which have played a crucial role in triggering geological disasters [28,34]. Nonetheless, in assessing the impact of human activities on such disasters, evaluations have frequently focused solely on proximity to infrastructure, such as roads. Incorporating land use changes as an additional evaluative criterion diversified and enhanced the consideration of human influences in the assessment process. Nonetheless, several limitations exist in this study. These include: (1) The dataset for geological disaster points collected in this new county study is insufficient; and (2) the methodology employed by the random forest model, which randomly selected samples not classified as disaster points, introduced a degree of uncertainty in accurately distinguishing potential disaster points.

## 7. Conclusions

1. An analysis utilizing the hierarchical analyzed method to assess the factors influencing geological disasters in Xinxian County identified distance from the road, average annual rainfall, distance from geological structure, and area of land use change as the primary determinants of geological disasters in the area. The increase in human engineering activities, driven by economic and social development, and the irrational excavation of natural slopes has significantly contributed to slope instability, thereby heightening the risk of geological disasters;
2. Comparative analysis between two models indicated similarities in predicting susceptibility to geological disasters within the study area. However, the AHP-informative model demonstrated superior accuracy and assessment rationality over the random forest model in evaluating susceptibility;
3. Geological hazards in Xinxian County were predominantly found in areas such as steep canyon banks and slopes, curved and concave banks of mountain rivers, watershed regions, eroded banks and slopes, and cliffs, as well as artificially steepened mountain slopes. Zones with extremely high susceptibility and a high likelihood of such hazards were primarily located along the near east–west, near north–south, northwest, and northeast fracture groups, as well as nearing Lick Tai Mountain’s back slope, the Baimashan–Xizhangdian complex slope, and the vicinity of the Baiyunshan dome;
4. The probability of geological hazards, including landslides, avalanches, and mudslides, increased with the intensity and cumulative amount of precipitation. The stability of slopes in the surveyed area was compromised to varying extents by precipitation, with rainfall being a key triggering factor for these hazards;
5. The gneiss rock group exposed in the Xinxian area had a large area, and many disaster points throughout the group were distributed across all of the towns and villages in the county. Because the strength of gneiss changes greatly under the action of weathering, and different weathering degrees result in vastly different effects, the properties of rock and soil mass with different weathering degrees was shown to play a decisive role in slope stability.

## Figures and Tables

**Table 1:** All data sources and evaluation systems in the study area.

Factors	Types	Sources
Elevation	Raster data (12.5 m)	Calculation and extraction of GDEMV3 12.5 M based on ArcGIS 10.8 software
Slope	Raster data (12.5 m)	
Aspect	Raster data (12.5 m)	
Slope shape	Raster data (12.5 m)	
Topographic relief	Raster data (12.5 m)	
Distance from geological structure	Vector data	Extracted using ArcGIS 10.8 software
Rock group	Vector data	Field data on geological hazards
Distance from road	Vector data	Extracted using ArcGIS 10.8 software
Land use cover type	Raster data (10 m)	www.globallandcover.com (9 March 2024)
Area of land use change	Raster data (10 m)	10 M land use treatments were obtained using ArcGIS 10.8 software.
NDVI	Raster data (12.5 m)	Landsat 8 OLI_TIRS Satellite data, http://www.gscloud.cn/ (9 March 2024)
Average annual rainfall	Raster data (12.5 m)	http://www.geodata.cn (9 March 2024)
River network density	Raster data (12.5 m)	

**Table 2:** Evaluation factors AHP weights.

Object Layer	Factor Layer	Index Layer	**w**
Geologic hazard susceptibility	Topography and geomorphology (U_1_)	Topographic relief (U_11_)	0.0181
		Slope aspect (U_12_)	0.0155
		Gradient (U_13_)	0.0217
		Slope shape (U_14_)	0.0091
	Geologic conditions (U_2_)	Rock group (U_21_)	0.0627
		Distance from geological structure (U_22_)	0.1254
	Human engineering activities (U_3_)	Distance from road (U_31_)	0.2838
		Area of land use change (U_32_)	0.0946
	Meteorology and hydrology (U_4_)	Annual average precipitation (U_41_)	0.1726
		River network density (U_42_)	0.0575
	Plant cover factor (U_5_)	NDVI (U_51_)	0.0463
		Land use (U_52_)	0.0926

**Table 3:** The amount of information graded for each evaluation factor.

Evaluation Factor	Classification	Disaster Points	Volume of Information
Slope shape	Concave slope	11	−0.31
	Linear slope	18	−0.10
	Convex slope	50	0.12
Rock group	Soil	9	1.59
	Hard and relatively hard quartz schist	4	−0.21
	Hard intrusive rock group Helatively hard massive	53	−0.04
	gneiss rock group	13	−0.27
Topographic relief	0~5	26	−0.39
	5~10	43	0.14
	10~15	9	0.69
	>15	3	2.13
Distance from geological structure	0~500	27	0.85
	500~1000	11	0.02
	1000~1500	7	−0.28
	>1500	34	−0.33
Slope	0~8.54	17	−0.21
	8.54~12.13	24	0.64
	12.13~17.73	27	0.36
	17.73~54.07	11	−0.88
NDVI	0~0.54	26	1.53
	0.54~0.62	18	0.62
	0.62~0.73	26	−0.31
	0.73~1	9	−1.15
Distance from road	0~102.2	56	0.45
	102.2~202.2	21	0.03
	>202.2	2	−2.44
Aspect	Flat	2	0.02
	N	3	−1.05
	NE	8	−0.13
	E	10	0.03
	SE	12	0.16
	S	9	−0.01
	SW	12	0.24
	W	15	0.40
	NE	8	−0.30
Land use cover type	Water	0	0.00
	Forest	27	−0.89
	Cropland	1	−1.78
	Impervious	51	2.09
Area of land use change	0~6.1533	66	−0.09
	6.1533~40.248901	7	1.22
	40.248901~86.414398	6	0.31
Average annual rainfall	<925	4	−0.25
	925~950	28	0.28
	950~975	3	−1.09
	975~1000	29	0.16
	>1000	15	−0.24
River network density	<0.00267	66	−0.08
	0.00267~0.080	5	0.64
	>0.008	8	0.51

**Table 4:** Hyperparameters of random forest.

Types	Parameters
N estimators	1000
Max depth	5
Criterion	Gini

**Table 5:** Statistics on the results of the AHP-information quantity model of susceptibility partitioning.

Geologic Hazard Susceptibility Zoning	Number of Disasters	Number of Slope Units	Slope Unit Area/km^2^	Area Proportion/%	Density of Disaster Sites/pcs/km^2^
Very High susceptibility area	33	678	193.15	12.43%	0.1709
High susceptibility area	24	945	366.02	23.56%	0.0656
Moderate susceptibility area	15	1467	607.32	39.09%	0.0247
Low susceptibility area	7	946	386.97	24.91%	0.0181

**Table 6:** Random forest model susceptibility partitioning results in statistics.

Geologic Hazard Susceptibility Zoning	Number of Disasters	Number of Slope Units	Slope Unit Area/km^2^	Area Proportion/%	Density of Disaster Sites/pcs/km^2^
Very High susceptibility area	30	598	194.45	12.52%	0.1543
High susceptibility area	21	966	336.39	21.65%	0.0624
Moderate susceptibility area	19	1560	635.34	40.90%	0.0299
Low susceptibility area	9	912	387.28	24.93%	0.0232

**Table 7:** Classification of engineering geologic rock groups and development of geologic hazards and hidden hazards.

Rock Group	Number of Geologic Hazard Sites
Soil	9
Hard, relatively hard quartz schist	4
Hard intrusive rock group	53
Helatively hard massive gneiss rock group	13

## Data Availability

Authors confirm that all relevant data of the present research are included in the article.
